# A New Journal

**Published:** 1881-11

**Authors:** 


### A NEW JOURNAL.

"THE DENTAL RECORD, a monthly *Journal of Dental Science, Art and Literature, devoted to the interests of the profession.*"

Edited by Thos. Gaddis, L. D. S., London, Eng.

This is a neat and well filled journal of 48 pages, issued monthly, published by the Dental Manufacturing Company, No. 25 Broad St.

In the preface the editor says, "The educational advancement of a body is always associated with the development of its literature. The *Dental Record* is the outcome of this advancement, and necessitated by the spreading influence of Dentistry as a Profession. It will offer every facility for the publication of matters of interest to the profession, and thus give a stimulus to men to write, and communicate the results of their research and experiences, and their opinions." Thus it will be seen that the *Record* is preparing for itself a large sphere of usefulness. There is, doubtless, room for this new journal. It should have a wide circulation, not only in European countries, but in the United States as well. With such contributors as Coffin, Coles, Elliott, Parkinson, Rogers, Stockton, Thompson, Walker and Weiss, it cannot be otherwise than interesting and instructive. Let it have a wide circulation.

